# Genotyping of non-polio enteroviruses associated with acute flaccid paralysis in Thailand in 2013 and 2014

**DOI:** 10.1186/s12985-021-01621-0

**Published:** 2021-07-23

**Authors:** Napa Onvimala, Nathamon Kosoltanapiwat, Pornpan Pumirat, Muthita Vanaporn, Suchitra Nimmanitya, Ratana Tacharoenmuang, Ratigorn Guntapong, Pornsawan Leaungwutiwong

**Affiliations:** 1 Department of Microbiology and Immunology, Faculty of Tropical Medicine, Mahidol University, Bangkok, Thailand; 2 Department of Medical Sciences, National Institute of Health, MOPH, Nonthaburi, Thailand; 3 Department of Disease Control, Bureau of General Communicable Diseases, MOPH, Nonthaburi, Thailand

**Keywords:** Acute flaccid paralysis (AFP), Non-polio enteroviruses (NPEVs**)**, Enterovirus (EV), Coxsackievirus (CA)

## Abstract

**Background:**

Acute flaccid paralysis (AFP) surveillance was conducted as part of the World Health Organization’s strategy for completely eradicating poliomyelitis and leaving non-polio enteroviruses NPEVs as one of the main potential causes of AFP. We aimed to detect NPEV in association with AFP.

**Methods:**

We used 459 isolates reported to be Negative Polio and some NPEVs by the World Health Organization Polio Regional Reference Laboratory (Thailand), which had been obtained during polio surveillance programmes conducted in Thailand in 2013–2014. Of 459 isolates, 35 belonged to the genus Enterovirus by RT-PCR and genotyping by DNA sequencing.

**Results:**

This study found 17 NPEV genotypes, with 3, 13 and 1 belonging to enterovirus (EV) species A (EV-A), EV-B, and EV-C, respectively. The EV-A types identified included coxsackievirus A2 (CA2), CA4, and EV71, typically associated with hand, foot and mouth diseases. EV-B is the most prevalent cause of AFP in Thailand, while CA21 was the only type of EV-C detected. The EV-B species (13/35; 76.5%) constituted the largest proportion of isolates, followed by EV-A (3/35; 17.6%) and EV-C (1/35; 5.9%). For the EV-B species, Echovirus (E) 30 and CVB were the most frequent isolates. E30, CVB, E14, and E6 were considered endemic strains.

**Conclusion:**

NPEVs, e.g. CA4, are reported for the first time in Thailand. Despite some limitations to this study, this is the first report on the circulation patterns of NPEVs associated with AFP in Thailand. AFP surveillance has unearthed many unknown NPEVs and, the cases of death due to AFP occur annually. Therefore, it is important to study NPEVs in the wake of the eradication of poliovirus in the context of the continued incidence of paralysis.

## Background

Acute flaccid paralysis is a clinical syndrome, which refer to a symptom of paralysis or weakness. There is much cause of infectious and non-infectious. It can be cause by many diseases such as poliomyelitis, enteritis, myositis, meningitis and encephalitis. Poliovirus was the main cause of AFP can cause poliomyelitis. In March 2014, Thailand completely eradicated poliomyelitis and left non-polio enteroviruses as one of the main potential causes of AFP [1]. So, we aimed to highlight the role of NPEV in association with AFP start from the year before and after eradicate. Enterovirus is a genus belonging to the family Picornaviridae [2–4]. According to the classification, 12 species can cause disease in both humans and animals [5–9]. However, 4 species of Enterovirus A-D and 3 species of Rhinovirus causes disease in humans. Enterovirus E-L causes disease in animals. Enteroviruses A (EVA), B, C, and D, consist of 25, 63, 23, and 5 types respectively. Rhinoviruses A, B and C are composed of 80, 32, and 57 types, while some are pending classification [2, 3]. Currently studies on non-polio enterovirus are being conducted in many countries, but the enterovirus isolated varies in each country [10–17]. The AFP surveillance system available in each country can detect non polio enteroviruses some are rare types such as EVC95 EVC105 in Northern India, EVC99 and EVA120 in Nigeria [18, 19], so it is necessary to study the isolated enterovirus genotype as a source for further study in each country. In Thailand, the last wild poliovirus was reported in 1997 [20], and Thailand was declared polio-free in 2014. The detection of Enteroviruses in AFP patients plays an important role when an enterovirus is found in an AFP surveillance cluster outbreak. Over the years, the number of poliomyelitis cases has reduced worldwide, and the disease has been eradicated in some parts of the world, leaving NPEV as one of the main potential causes of AFP. Regarding NPEVs identified by AFP surveillance in Thailand in 2013 and 2014, almost 50% were untyped, based on WHO protocols for EV detection [21]. Many researchers found NPEVs in AFP patients [22–25]. EVA71 are a public health problem in Thailand and can lead to death. A study of the role of EVA71 in Brazil showed a resultant link to some AFP cases [26]. Therefore, we must be alert for NPEV in AFP cases. Against this background, identifying the genotypes circulating in the country may improve our understanding of the epidemiology of EV infection; moreover, in the wider context of polio eradication, it may provide assurance that Poliovirus is not being overlooked. Laboratory data were analysed to provide an overview of the NPEV genotypes identified and their occurrence, diversity, and patterns of circulation in cases of AFP identified in Thailand in 2013 and 2014.

## Methods

### Ethics approval

The samples in this study were the unidentified isolate from AFP surveillance at the RRL, National Institute of Health and discard non polio AFP by expert review committee from Department of Medical Science, Department of Disease Control and Bureau of Epidemiology, Ministry of Public Health, Nonthaburi, Thailand. So, we didn’t need an ethical approval to conduct the research.

### Virus isolated samples

**Fig. 1:**
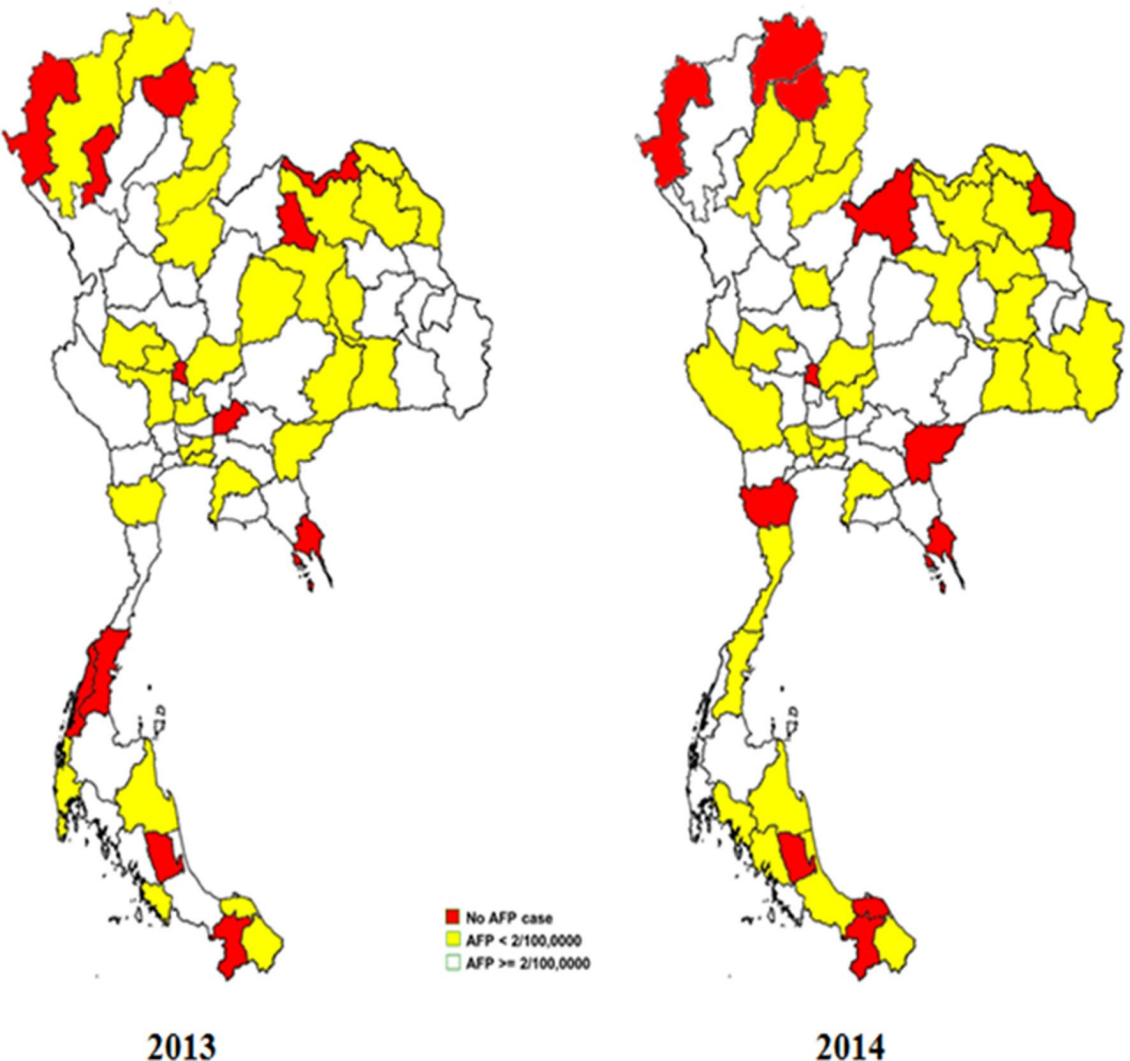
Acute flaccid paralysis (AFP) surveillance in 71 Thai provinces in 2013 and 2014. Red represents no AFP cases, whereas yellow and white represent AFP rates of < 2 or ≥ 2 per 100,000 children aged below 15 years, respectively

This study focused on a total of 459 isolates from patients with AFP aged < 15 years who were identified by AFP surveillance in Thailand in 2013 and 2014. The stool samples from 71 provinces were sent to the WHO Polio Regional Reference Laboratory (RRL) for polio screening at the National Institute of Health, Department of Medical Science, Ministry of Public Health, Thailand (Fig. 1). The samples were isolated in RD and L20B for detected polio. This step was done by RRL of Thailand. All of them re-inoculated in RD, Hep2 and Vero for increase the chance to isolate more NPEV.

### RNA extraction and RT-PCR typing

In this study, we confirmed the status of NPEV using reverse transcription polymerase chain reaction (RT-PCR). Total RNA was extracted from positive isolated of stool samples stored at -20 degree Celsius using QIAamp Viral RNA Extraction Kit (Cat. No. 52906; Qiagen). Among these unidentified isolates, 24 NPEVs (5.2%) were isolated in 2013 and 2014 and were confirmed to be NPEVs by performing viral culture within 14 days at RRL; finally, 11 NPEV isolates were re-inoculate in more cell culture RD, Vero, Hep2, GL-37 the positive was show CPE within 14 days we observed CPE under microscope and used all positive for analysis in this study. 35 positive isolates on analysis using pan-EV primers VP1 region of the Enterovirus surveillance guidelines. The typing was not performed on all RD-positive isolates as per the timeframe set by the WHO, according to which the result must be released within 14 days of receipt of specimens. This study was not part of the routine surveillance programme based on the typing of all NPEV isolates; therefore, we identified EVs using universal pan-EV primers with RT-PCR specific to the viral polyprotein 1 (VP1) region as universal primers of EV [27]. We used a two-step RT-PCR. The first step for cDNA amplification by using four primers which were specific to enterovirus group A B C and D then performed the second step of PCR for pan enterovirus VP1 region (Table 1).

**Table 1:** Primers used for cDNA synthesis and PCR amplification

cDNA (RT) Primers	Sequence
Enterovirus group A	AN32 5′ GTY TGC CA 3′
Enterovirus group B	AN33 5′ GAY TGC CA 3′
Enterovirus group C	AN34 5′ CCR TOR TA 3′
Enterovirus group D	AN35 5′ RCT YTG CCA 3′

PCR primers	Sequence
SO224F	5′ GCI ATG YTI GGI ACI CAY RT 3′
SO222R	5′ C ICC IGG IGG IAY RWA CAT 3′

### VP1 sequencing

Further, genotypes were analyzed by DNA sequencing. The purified PCR products were sent for chain-termination DNA sequencing (Macrogen, Seoul, Korea). The nucleotide sequences were aligned with reference sequences of EV genotypes obtained from the GenBank database using ClustalW in BioEdit version 7.2.6.1. The phylogenetic tree was constructed with the maximum likelihood and Bayesian methods with 1,000 bootstrap replicates using the MEGA 7 software.

## Results

**Fig. 2:**
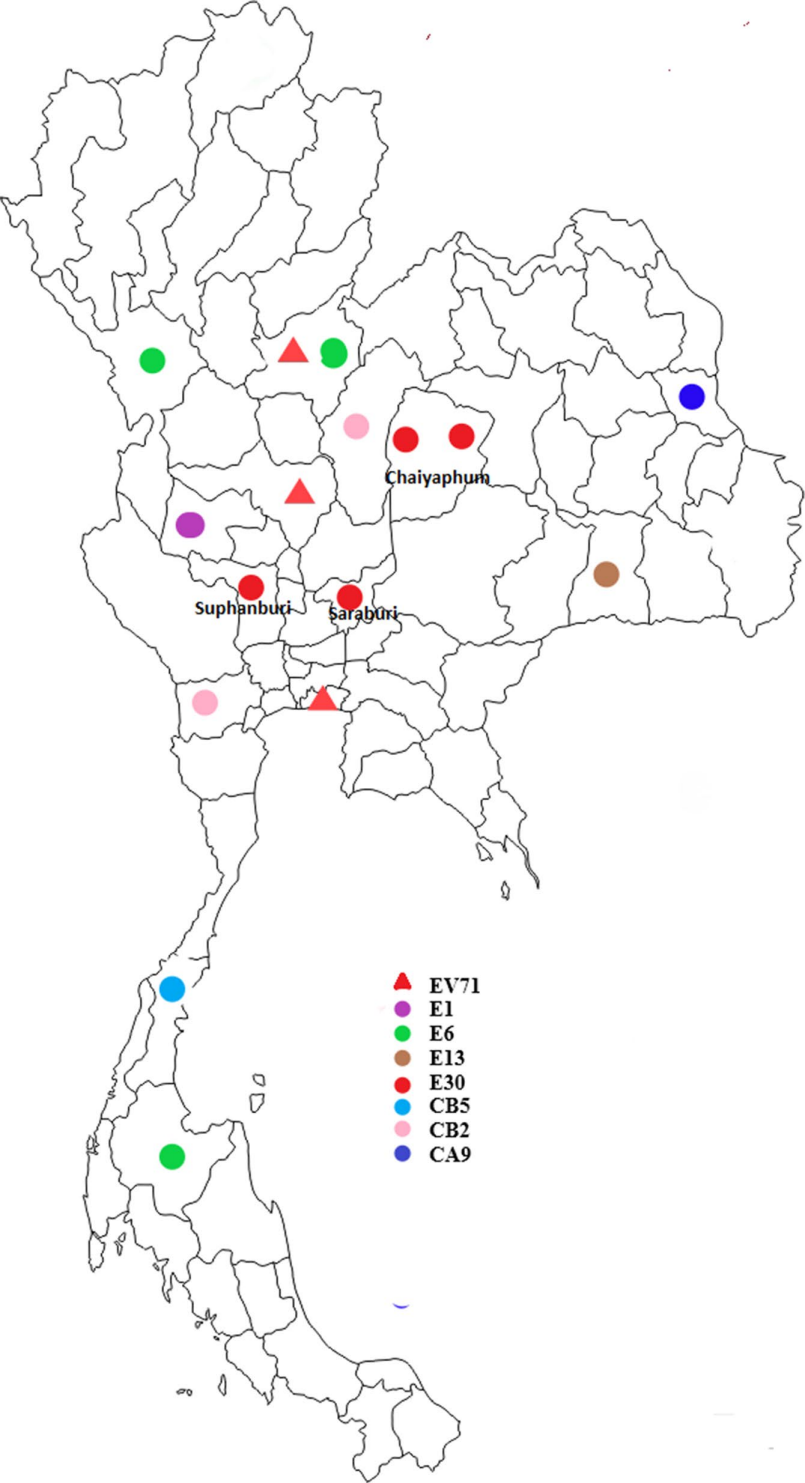
Genotype distribution of enteroviruses in Thailand in 2014. Triangle represents EVA, whereas circle represents EVB, red triangle: EV71, purple circle: Echo1, green circle: Echo6, blown circle: Echo13, red circle: Echo30, yellow circle: CB5, pink circle: CB2 and blue circle: CA9

All 459 originally unidentified isolates isolated from RD cells and reported to be NPEVs were confirmed to be EVs by RT-PCR specific to the VP1 region. Viral RNA was extracted from the unidentified isolates using four primers specific to EV groups A, B, C and D, as described previously [27]. The 35 isolates which were found to be positive on RT-PCR were genotyped by DNA sequencing targeting the VP1 region. In this study, we identified 17 genotypes of NPEVs in AFP and classified them into three major groups, namely, EV-A, EV-B and EV-C. EV capsid gene (VP1) sequences were amplified using a previously described PCR protocol using the primers SO224F and SO222R, leading to the generation of fragments of approximately 670-bp length [27]. RT-PCR facilitated the identification of 7.6% (n = 35) of NPEVs and negative isolates, most of which belonged to EV-B. For the remaining isolates (n = 12), no positive results were obtained by RT-PCR and considered untypable EVs. The viral genomes of the 35 isolates were positive for the VP1 region on RT-PCR. The VP1 sequences were compared with those obtained from GenBank and were assigned the genotype of the strain which had the highest identity score. The sequences were classified into genotypes when the homology of the VP1 sequence to prototype strains was at least 90%. The results of genotyping were summarised in Table 2. A cluster of patients with AFP in Chaiyaphum Province in June 2014 who were found to be positive for Echo30 with one dead case, AFP cases from Saraburi Province with the onset of paralysis were found to be positive in the same month. (Fig. 2).

**Table 2:** Enterovirus genotypes from 35 non-polio enteroviruses in Thailand in 2013 and 2014

	EV-A			EV-B													EV-C
Year	CA2	CA4	EV71	E1	E6	E11	E13	E14	E18	E20	E30	CB1	CB2	CB3	CB5	CA9	CA 21
2013	2	1	–	–	–	2	1	4	1	1	1	1	0	1	–	–	4
2014	–	–	3	1	3	–	1	–	–	–	4	–	2	–	1	1	–
Total	2	1	3	1	3	2	2	4	1	1	5	1	2	1	1	1	4

CA, Coxsackievirus A; EV, Enterovirus; E, Echovirus; CB, Coxsackievirus B

**Fig. 3:**
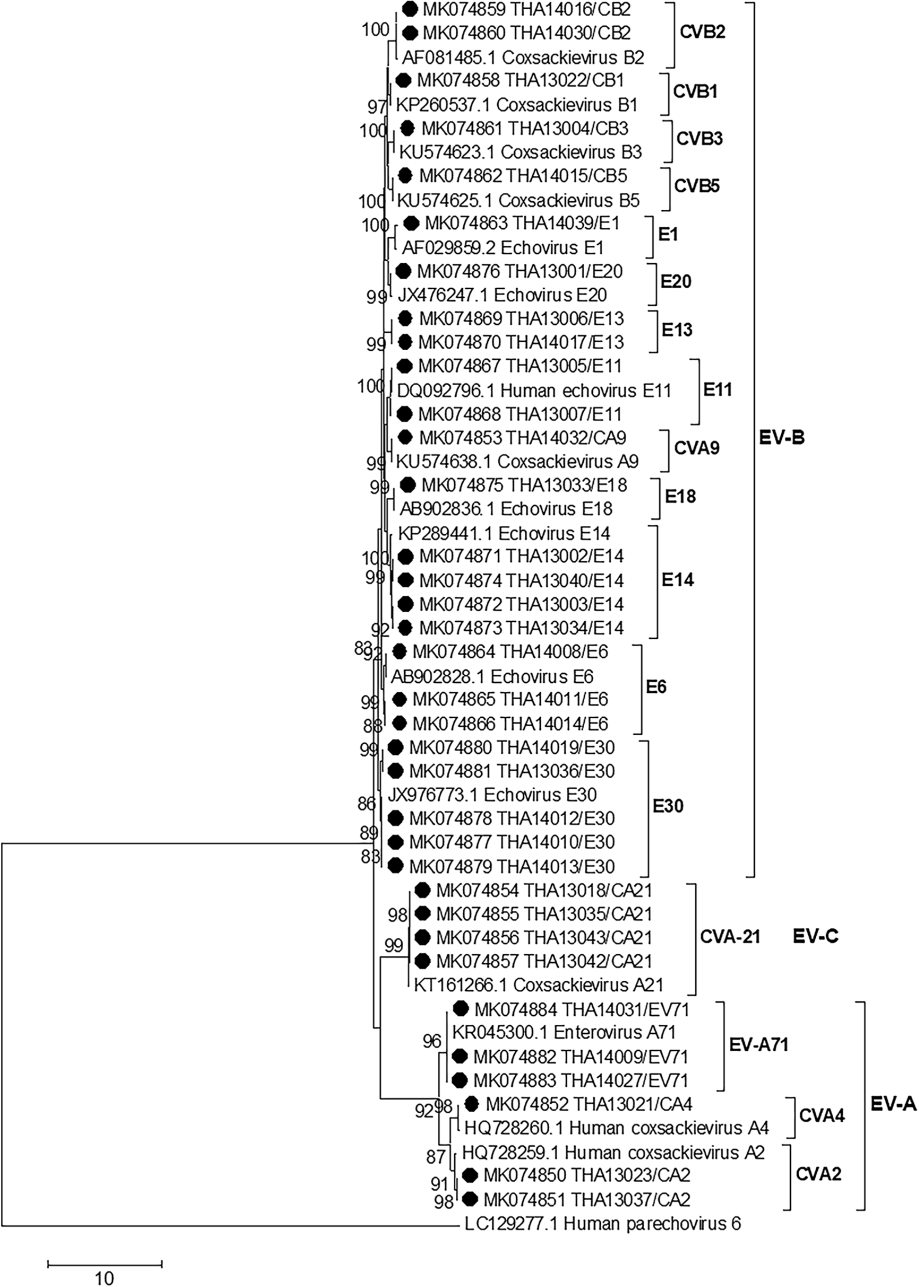
Phylogenetic analysis of partial viral polyprotein 1 nucleotide sequences. The phylogenetic tree was inferred using the maximum likelihood method based on the general time reversible model and a bootstrap value of 1000. Virus names and accession numbers obtained in this study are marked with black circles. The sequence of human parechovirus 6 was used as outgroup. Bootstrap values > 80 is indicated at nodes. Scale bar represents nucleotide substitutions per site

Based on the distribution of NPEV types in 2013 and 2014, E30 and CB were found to be the predominant genotypes, followed by E14, CA21, E6 and EV71. Notably, the data from Table 2 showed that the circulation pattern no EV-D-type isolates was identified to be endemic. EV-B predominantly circulated as an endemic type, but EV-C showed only one type, CA21. These have never previously been reported in Thailand. Partial VP1 sequence data revealed that 7.6% of the 35 NPEV isolates could be typed using RT-PCR and nucleotide sequencing (Fig. 3).

## Discussion

NPEVs have been detected in AFP cases worldwide, but they can cause a wide range of clinical diseases such as viral myositis, hypokalaemia, and encephalitis [28, 29]. The most prevalent type of NPEV in Thailand was found to be EV-B. EV71, an EV-A, is regarded as a major causative agent of hand, foot and mouth disease (HFMD) [30]. Roberts et al. also detected EV71 in AFP [31]; they revealed that an EV71-based outbreak occurred along the Australian coast in 2013. Two deaths associated with EV71 infection were reported, and the sub-genogroup C4a was isolated by virus culture or detected by RT-PCR from stool specimens of nine AFP cases. Moreover, additional three non-polio AFP cases were associated with EV71 based on the clinical evidence, representing 20% of the AFP cases with the onset of paralysis in 2013 [31]. One patient was admitted to a hospital for high fever, and an endotracheal tube was used. She suffered a symmetric paralysis (motor power grade I) and died within 1 week of the onset of paralysis. Anyway, we could not collect the stool sample from the death case, then we collect the sample from close contact person and can identify CA4 from the isolate of close contact person of this death case. Further, Hu et al. (2011) reported that CA2, CA4, CA5 and CA10 were isolated from patients with HFMD during an outbreak in China in 2009. EV-B was the variant most frequently identified in their study [32]. In this study, Echo30 was the most prevalent type which was widely distribution in Thailand. There have been many reports on large outbreaks of aseptic meningitis being caused by Echo30 from many parts of the world, such as in the United States [29] and two cities in Turkey [33]. CA21 was the only genotype of EV-C found in our study from two isolates from Nakhon Ratchasima in January and May 2013. The other isolates were identified in SamutPrakarn and Si Sa Ket in March and August 2013, respectively. One of four isolates which could be identified was from an AFP case in Nakhon Ratchasima resulting in death in May 2013. Zichun et al. found that two rare human EV types, CA21 and EV68, are detected most frequently in human EV-positive adults with acute respiratory infections [30]. In 2019, (The Annual Medical Sciences Conference 27^th^) RRL show the most prevalent type which was distribution in Thailand were EV-B and EV-A. They could not detect EV-C and EV-D. The most type of EV-B is Echo11 and CoxB2. They also found 2 EV71 in EV-A are mix infection with Echo6 from EV-B. They are the major causative agents of hand, foot and mouth disease. So, the most prevalent type of NPEV after Polio eradicate from Thailand were EV-B and with a continued incidence of AFP, NPEV should continue to be detected.

## Conclusions

The information of NPEV presented in this paper is valuable. Samples of CA4, Echo30 and CA21 identified which were obtained from patients who died of AFP should be shared and the complete sequences should be studied to identify the strains and to shed light on the virulent strains currently circulating in Thailand. AFP surveillance has unearthed many unknown isolates and cases of death due to AFP occur annually. Therefore, it is important to study other enteroviruses in the wake of the eradication of poliovirus in the context of the continued incidence of paralysis. Studies can be conducted annually to investigate the types of NPEV, especially unidentified ones, in future and to establish diseases in surveillance systems that target NPEVs in the country.

## Data Availability

All data generated or analyzed during this study are included in this manuscript and Genbank.

## Abbreviations

AFP: Acute flaccid paralysis
NPEV: Non-polio enteroviruses
CA: Coxsackievirus A
CB: Coxsackievirus B
E: Echovirus
EV: Enterovirus
EVA: Enterovirus A
EVB: Enterovirus B
EVC: Enterovirus C
EVD: Enterovirus D
HFMD: Hand, foot and mouth disease
RD: Rhabdomyosarcoma
RT-PCR: Reverse-transcriptase polymerase chain reaction
VP1: Viral protein 1
